# Effect of Humidity and Temperature on the Impedances and Voltage of Al/Gr-Jelly/Cu-Rubber Composite-Based Flexible Electrochemical Sensors

**DOI:** 10.3390/gels8020073

**Published:** 2022-01-24

**Authors:** Muhammad Tariq Saeed Chani, Khasan S. Karimov, Esraa M. Bakhsh, Mohammed M. Rahman

**Affiliations:** 1 Center of Excellence for Advanced Materials Research, King Abdulaziz University, P.O. Box 80203, Jeddah 21589, Saudi Arabia; mmrahman@kau.edu.sa; 2 Chemistry Department, Faculty of Science, King Abdulaziz University, P.O. Box 80203, Jeddah 21589, Saudi Arabia; tariqchani1@gmail.com; 3 Ghulam Ishaq Khan Institute of Engineering Sciences and Technology, Topi 23640, Pakistan; khasan@giki.edu.pk; 4 Center for Innovative Development of Science and Technologies, Academy of Sciences, Rudaki Ave., 33, Dushanbe 734025, Tajikistan

**Keywords:** graphene, jelly, superabsorbent polymers, graphene–jelly electrolyte composite, shockproof flexible sensor, drop casting, short-circuit currents

## Abstract

Here we present the fabrication of graphene and jelly (superabsorbent polymer) electrolyte composite-based shockproof flexible electrochemical sensors (Al/Gr-Jelly/Cu) and their properties under the effect of humidity and temperature. A layer of graphene mixed in jelly electrolyte was drop-casted onto porous rubber substrates between preliminary fixed aluminum (Al) and copper (Cu) electrodes followed by rubbing-in. It was observed that the graphene and jelly mixture was mechanically soft and flexible, similar to jelly. Electrically, this mixture (graphene and jelly) behaved as a flexible electrolyte. It was observed that under the effect of humidity ranging from 47 to 98%, the impedances of the sensors decreased by 2.0 times on average. Under the effect of temperatures ranging from 21 to 41 °C the impedances decreased by 2.4 times. The average temperature coefficient of impedances was equal to −0.03 °C^−1^. The electrochemical voltage generated by the flexible jelly electrolyte sensors was also investigated. It was found that the initial open-circuit voltages were equal to 201 mV and increased slightly, by 5–10% under the effect of humidity and temperature as well. The short-circuit currents under the effect of humidity and temperature increased by 2–3 times. The Al/Gr-Jelly/Cu electrochemical sensors may be used as prototypes for the development of the jelly electronic-based devices.

## 1. Introduction

As is known, humidity sensors are used in different areas of modern technology and their properties have been described in detail in the literature. Ref. [1] presented a review of materials and mechanisms for fabrication of the humidity sensors. The principles, mechanisms, and fabrication technologies of the humidity sensors were presented in Ref. [2]. The description of the thin films-based organic resistive and capacitive humidity sensors was given in Ref. [3]. The graphene oxide and PEDOT:PSS methyl red-based humidity sensors showed full range detectability and high sensitivity [4]. The synthesis and characterization of amino anthraquinone and its application as an active material in environmental sensors were described in Ref. [5].

For environmental monitoring the organic semiconductor copper phthalocyanine-based illumination and humidity sensors and their properties were described in Ref. [6]. The properties of organic–inorganic composite-based sensors were also described in Ref. [7]. A number of research articles related to humidity sensors were published by our group as well. The humidity sensing, microstructural, and phase properties of the cuprous-oxide/orange dye composite were discussed in Ref. [8]. The graphene–orange dye solid electrolyte-based cells were characterized for humidity sensing [9]. The properties of the elastic rubber–graphene composite-based multi-functional sensors fabricated by using the rubbing-in technique were studied in Ref. [10].

During the past few years, number of graphene-based electronic devices have been designed and fabricated. The graphene-based electrochemical sensor and biosensors were fabricated and investigated by Shao et al. [11]. In Ref. [12] the wearable graphene-based infrared photodetector and temperature sensors were fabricated on a flexible polyimide substrate. A reduced graphene oxide-based flexible temperature sensor was designed and tested by Liu et al. [13]. A graphene/graphene-oxide hybrid membranes-based temperature sensor was fabricated and tested by Sun et al. [14]. The graphene-based electrochemical sensors and biosensors were designed, fabricated, and investigated as well [11].

Moreover, a number of electrochemical sensors were designed, fabricated, and investigated based on graphene as well. The electrochemical sensors fabricated on paper (where the paper was used as a scaffold to create electrodes of porous carbon nanotubes) were investigated in Ref. [15]. The conjugated polymers-based electrochemical sensors were described in Ref. [16]. Nanocomposite hydrogels for biomedical applications were described in Ref. [17]. 

Table 1 presents the overview of previous related work on sensor types, active materials, and fabrication techniques. 

In pursuance of our work on the sensors, in this paper we are presenting the data about the fabrication and investigation (of the electric properties) of Al/Gr-Jelly/Cu-rubber composite-based shockproof flexible solid electrochemical humidity and temperature sensors. 

## 2. Results and Discussions

**Figure 1:**
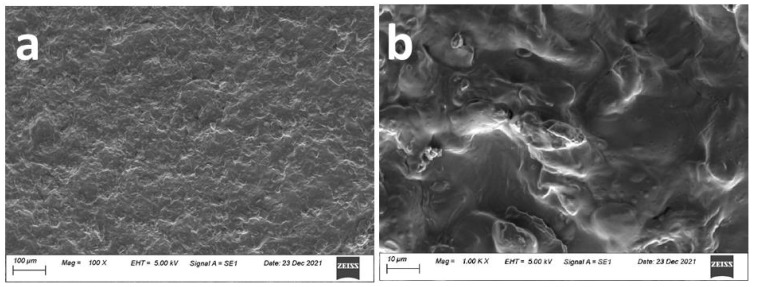
The SEM images showing the surface morphology of the graphene–jelly coating on the rubber substrate at (**a**) low and (**b**) high magnifications. The surface roughness is more pronounced in the micrograph.

Figure 1a shows the graphene–jelly coating done manually using a metallic load that moved in a horizontal plane, thus pressing it against the rubber surface. The coating had a rough appearance with the pressed material oriented along one of the directions, revealing the shear forces present during the application. The coating was continuous with occasional porosity, and the pores did not seem to penetrate to the rubber substrate. The continuity of the layer revealed in the micrographs indicated that the properties of the coating were uniform within the layer and only when the layer was stretched would there be a change in properties such as the electrical conductivity for pressure sensing, etc. Figure 1b shows the image at a high magnification. The surface roughness is more pronounced in the micrograph, which is required for the higher sensitivity of the devices.

**Figure 2:**
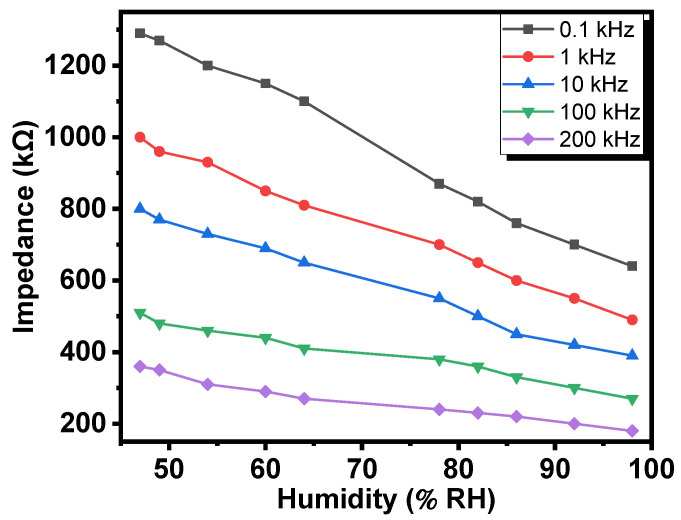
Dependences of the impedances of the Al/Gr-Jelly/Cu electrochemical jelly sensor on the relative humidity.

Figure 2 shows the dependences of impedance of the Al/Gr-Jelly/Cu-rubber composite-based electrochemical sensor on the relative humidity at 22 °C (constant temperature). It was seen that on increasing humidity (from 47 to 98% *RH*) the impedance decreased: in particular, 2.02 times (at 100 Hz), 2.04 times (at 1 kHz), 2.05 times (at 10 kHz), 1.89 times (at 100 kHz), and 2.0 times (at 200 kHz). It was also seen that on increasing the frequency the impact of humidity on the impedance or on the sensor was approximately the same. On average, the change of the impedances was equal to 2.0. The influence of humidity on the properties of the sensor was estimated by the humidity coefficient of impedance (*HCI*):

*HCI* = Δ*Z*/Δ*RH* (1)

where Δ*Z* is the impedance change with the change in humidity (relative) (Δ*RH* in %). By calculation it was found that the average *HCI* is equal to 1.32 kΩ/%. 

**Figure 3:**
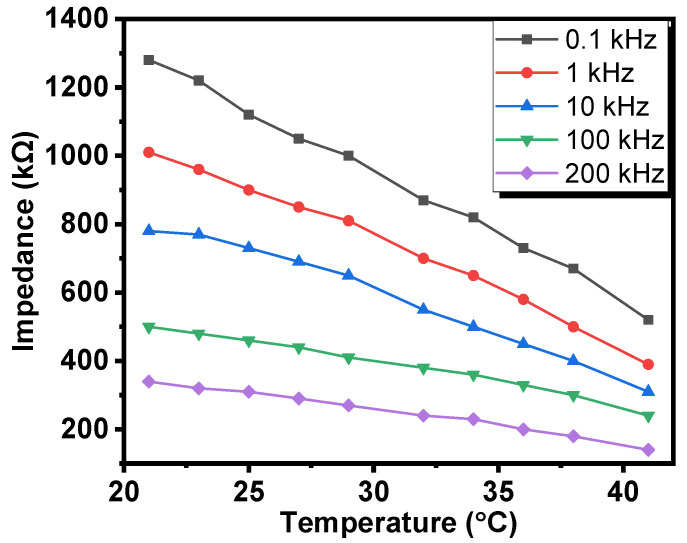
Dependence of the impedances of the Al/Gr-Jelly/Cu electrochemical sensors on temperature.

Figure 3 displays the dependence of the impedance of Al/Gr-Jelly/Cu-rubber composite-based electrochemical sensors on the temperature (in the range of 21 to 41 °C) at ambient humidity (47% RH). The increase in temperature caused the impedance to decrease, in particular: 2.46 times (100 Hz), 2.59 times (1 kHz), 2.52 times (10 kHz), 2.08 times (100 kHz), and 2.43 times (200 kHz). It was seen that with the increase in frequency the influence of temperature on the impedance of the sensor was approximately the same; on average, the change of impedances was equal to 2 times on changing temperature from 21 to 41 °C.

Analogous to the temperature coefficient of the resistance (*TCR*), the temperature coefficient of impedance (*TCI*) can also be introduced:

*TCI* = Δ*Z*/*Z*Δ*T*. (2)

The values of the temperature coefficient of impedance (*TCI*) were equal to (−0.030) °C^−1^ for 0.1 kHz, (−0.031) °C^−1^ for 1 kHz, (−0.030) °C^−1^ for 10 kHz, (−0.026) °C^−1^ for 100 kHz and (−0.026) °C^−1^ for 200 kHz.

Comparison of these values of the temperature coefficient of impedance with the *TCR* of some of the metals that are used in electronics (e.g., silver (0.0038), copper (0.0039) and aluminum (0.0043)) showed that the resistance-temperature behavior of the graphene–jelly composite was similar to that of semiconductors rather than that of metals. 

**Figure 4:**
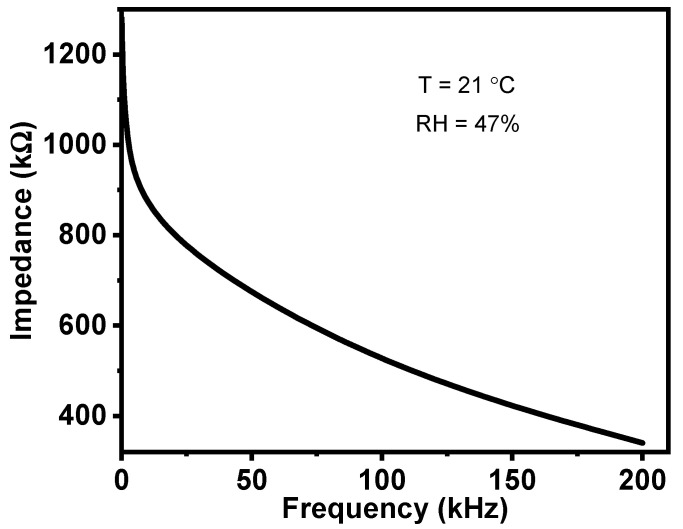
Dependence of the impedance of the Al/Gr-Jelly/Cu-rubber electrochemical sensor on frequency.

Figure 4 shows the impedance–frequency relationship of the Al/Gr-Jelly/Cu-rubber composite-based electrochemical sensors at a temperature of 21 °C and a humidity of 47% RH. It can be seen that on increasing the frequency the impedance decreased. This can be explained by the presence of “built-in” resistance and capacitance that can be shown in the sensor’s equivalent circuit.

**Figure 5:**
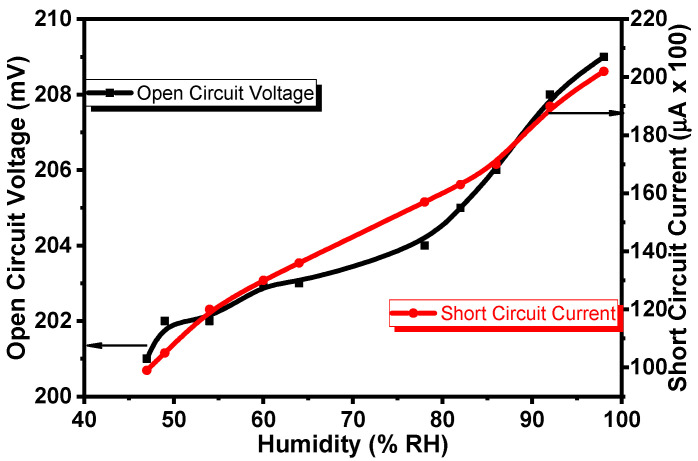
Dependence of the *V_oc_* (open-circuit voltage) and *I_sc_* (short-circuit current) of the Al/Gr-Jelly/Cu electrochemical sensor on humidity.

It was found that the fabricated Al/Gr-Jelly/Cu electrochemical sensors generated voltage. Figure 5 shows the dependences of the *V_oc_* (open-circuit voltage) and *I_sc_* (short-circuit current) of the sensor on humidity. The *V_oc_* increased by 5% and *I_sc_* increased by 2 times on increasing the relative humidity from 47 to 98%.

**Figure 6:**
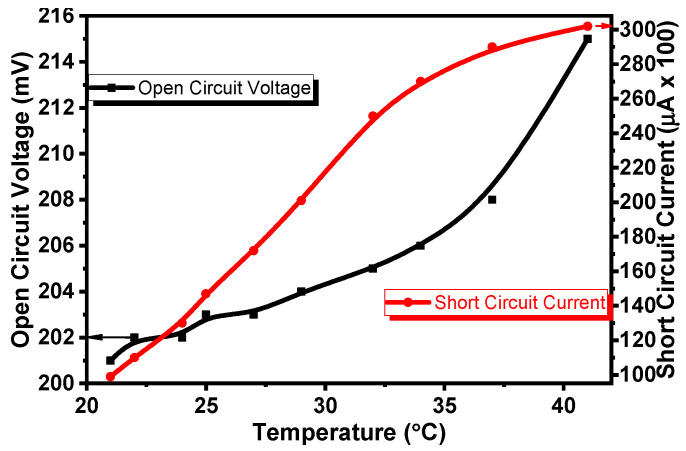
Dependence of *V_oc_* (open-circuit voltage) and the *I_sc_* (short-circuit current) of the Al/Gr-Jelly/Cu electrochemical sensor on temperature.

Figure 6 shows the dependences of the *V_oc_* and *I_sc_* of the Al/Gr-Jelly/Cu electrochemical sensor on temperature. It was seen that the *V_oc_* and the *I_sc_* of the sensor depended on the temperature as well (Figure 6): on increasing the temperature from 21 to 41 °C the *V_oc_* increased by 7% and the *I_sc_* increased by 3 times. 

**Figure 7:**
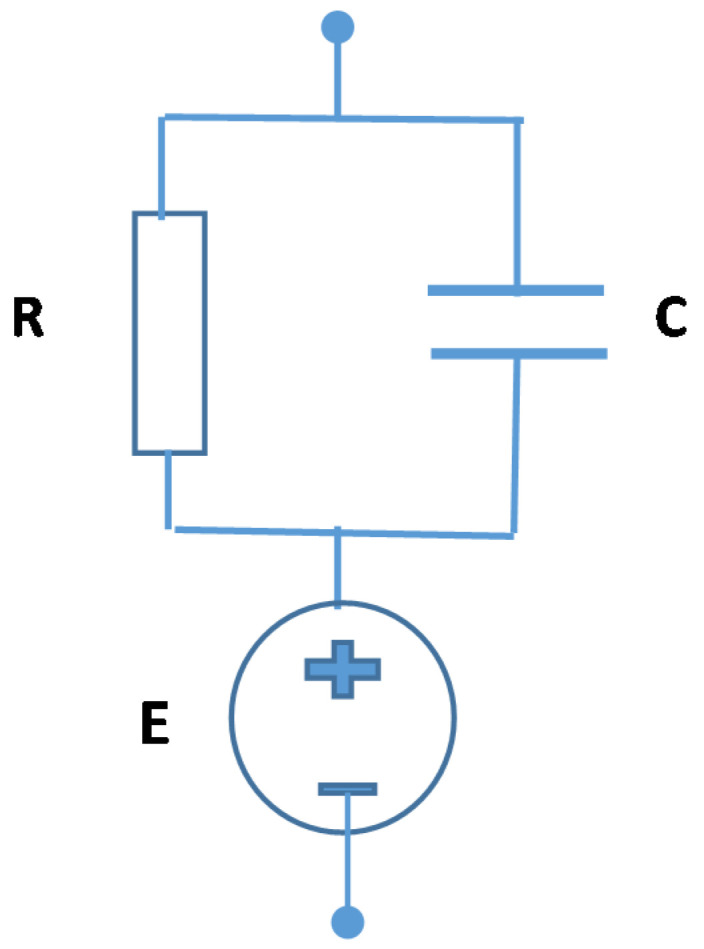
Simplified equivalent circuit of the Al/Gr-Jelly/Cu electrochemical sensor.

The equivalent circuit of the Al/Gr-Jelly/Cu electrochemical sensor is shown in Figure 7, which is a parallel connection of the resistance *R* and the capacitance *C*, while the voltage source *E* is connected in series. The fabricated electrochemical sensors have two metallic electrodes with different standard electrochemical potentials equal to −1.66 V for Al and +0.34 V for Cu; that is why the generation of the electric voltage takes place [19,20].

**Figure 8:**
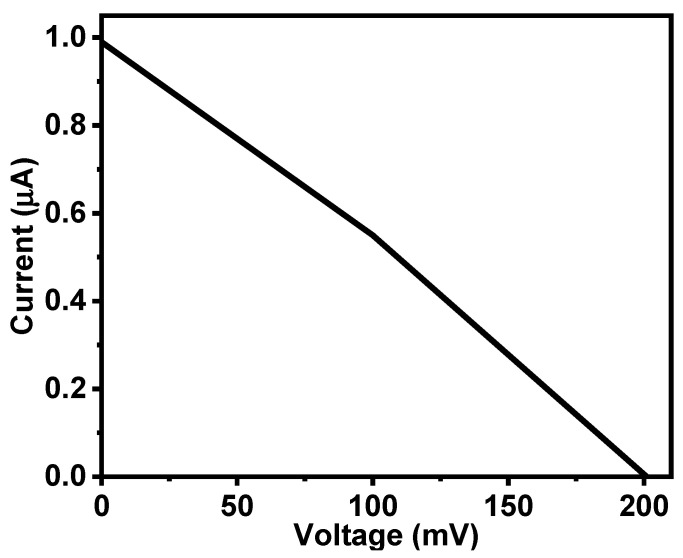
I–V characteristics of the Al/Gr-Jelly/Cu electrochemical sensors at 47% humidity and 21 °C temperature.

Figure 8 shows the I–V characteristics of the Al/Gr-Jelly/Cu-rubber electrochemical sensor at a humidity of 47% and a temperature of 21 °C.

The literature survey allowed disclosure of the following information concerning the investigations regarding the effect of humidity and temperature on the impedance and voltage of Al/Gr-Jelly/Cu-rubber composite-based flexible electrochemical sensors. Concerning the humidity effect on the electric properties of the materials two mechanisms may be considered. First of all, diffusion of water molecules into the material increases the dielectric permittivity. Secondly, the self-ionization of water molecules into protons (H^+^) and hydroxide ions (OH) finally results in the separation of (H^+^) and (OH^−^) (as shown in Equation (3)) and causes the concentration of charges to increase.

H_2_O <=> H^+^ + OH. (3)

In Ref. [1] the mechanisms of the humidity effect on the electric properties of the polymer materials were discussed as well. Moreover, the ceramic and polymer-based sensors were also reviewed. Sensitivity, response time, stability, and the sensing mechanism were also discussed. The literature survey allowed us to disclose the information concerning the investigations of the electrochemical sensors shown in this paper.

Concerning the effect of temperature on the electric properties of the organic materials, it should be said that there were several investigations. In particular, Ref. [21] investigated the I-V characteristics of the graphene samples, and it was found that the current especially increased when temperature reached above 150 °C.

The analysis of the data from the literature showed that the obtained results in this paper are supplementary. We hope these results will be useful, first of all for the fabrication of cheap and flexible devices, which may be especially important as a teaching aid. Secondly, these results are also important for the investigation and understanding of the physical and electrochemical properties of the flexible composites and their potential applications. In particular, the application of organic materials in applied electronic devices is very important, such as the realization of jelly electronics in vibration conditions.

## 3. Conclusions

In this paper information about the materials, fabrication technology, and the properties of environmentally friendly flexible electrochemical humidity and temperature sensors was presented. The sensor, having Al and Cu electrodes, was based on the organic material graphene and jelly composite as an active material. It was found that the sensor’s impedance was sensitive to the concentration of the humidity, temperature, and frequency of the applied voltages. Moreover, the sensor’s short-circuit current depended on the humidity and temperature. The sensor is a combination of three elements: resistance, capacitance, and electrochemical cells. First, the proper selection of the ingredients (graphene and jelly) and their ratio; second, the fabrication technology; and third, the structure of the sensors allowed us to realize the shockproof flexible humidity and temperature sensors. Because of the simplicity of fabrication technology these sensors can be used in practice and as a teaching aid as well. Our preliminary investigations and estimations showed that the sensors are not costly, having practically acceptable sensitivity and stability of the properties. Much graphene-related research work has shown that this organic material is environmentally friendly; no negative effect was observed by us as well. Our preliminary experiments showed that in this sensor the copper can be replaced by CNT (the work functions of both materials are close to ~5 eV), which can make it cheaper and acceptable for utilization in practice.

## 4. Experimental

**Figure 9:**
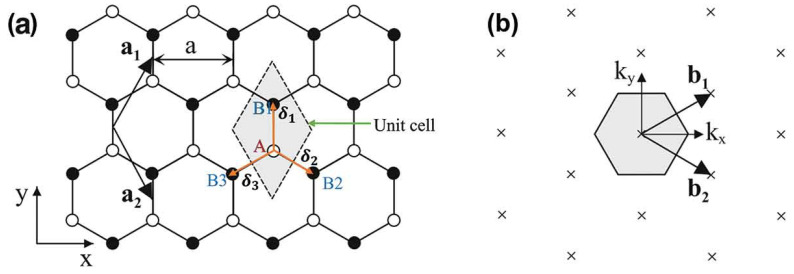
(**a**) Monolayer graphene (honeycomb lattice), where the carbon atoms are indicated by white (black) circles on A (B) sites, and (**b**) the monolayer graphene’s reciprocal lattice, where the corresponding Brillouin zone is indicated by shaded hexagon.

To fabricate the Al/Gr-Jelly/Cu-rubber composite-based flexible electrochemical sensor the graphene was purchased from the Sigma Aldrich (Merck KGaA, Darmstadt, Germany), while the jelly and the rubber substrates were purchased from the market. The graphene’s structure and its disorders were discussed in Ref. [22]. Figure 9 shows the molecular structures of the graphene.

The gel used for these sensors is made of superabsorbent polymers such as cross-linked sodium polyacrylate, cross-linked sodium carboxymethyl cellulose, etc. This gel is inodorous, non-edible, environmentally friendly, and used for fairy crafts for kids and indoor plantations. It is commercially available with a trade name of Rainbow Crystal Clay (http://www.miracle-chemical.com/Products.asp?ClassID=115, accessed on 6 December 2021) (Tianhe District, Guangzhou, China). The addition of other materials such as graphene, CNTs, or orange dye makes the gel conductive or semiconductive. Moreover, it is also important that the gel should be like glue.

**Figure 10:**
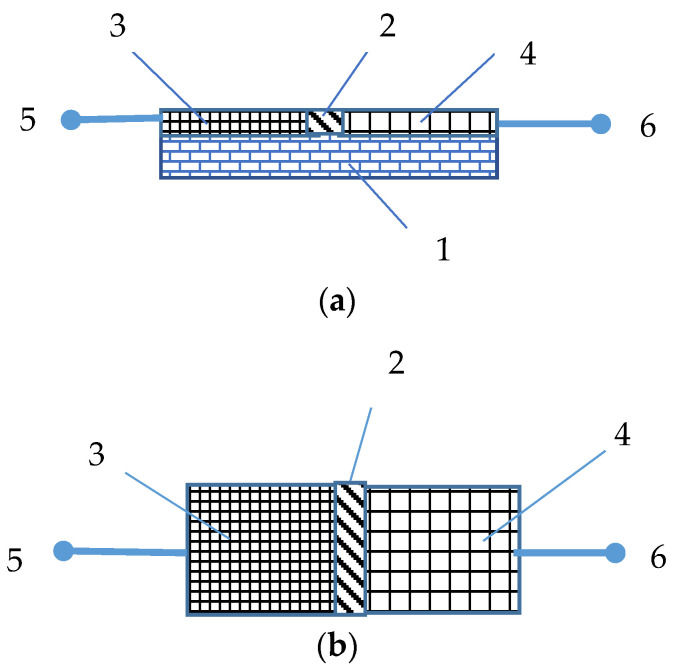
Schematic diagram showing the front view (**a**) and the top view (**b**) of the Al/Gr-Jelly/Cu-rubber composite-based shockproof flexible electrochemical humidity and temperature sensors: rubber substrate (1), graphene and jelly (superabsorbent polymer) composite (2), Al electrode (3), Cu electrode (4), metallic terminals (5 and 6).

The humidity and temperature sensors based on graphene/transparent jelly were fabricated by the rubbing-in technique. The mixture of ingredients was deposited onto rubber substrates between the preliminary fixed Al and Cu foil electrodes. In the Al/Gr-Jelly/Cu-based sensors, the graphene and jelly adhesive composite was in the following proportion: 50 wt. % and 50 wt. %. The gap between two (Al and Cu) electrodes was kept to 1 mm. The sizes of the rubber substrates were the following: length, width and thickness were equal to 20:10:10 mm^3^. Figure 10 illustrates the schematic of the front view (a) and the top view (b) of the Al/Gr-Jelly/Cu-rubber composite-based shockproof flexible electrochemical humidity and temperature sensor.

The micrographs were taken using an Evo-15 W filament scanning electron microscope at an accelerating voltage of 5 KeV. A secondary electrons detector was used to obtain the micrographs.

The MT 4090 (digital LCR-meter) was used for the measurement of the impedance at various frequencies ranging from 100 Hz to 200 kHz. For the measurements of the voltage the DT 4253 multimeter was used. Humidity and temperature were measured by the TECPEL 322. The testing was done in a special chamber with a built-in heating system.

## Figures and Tables

**Table 1:** Overview of previous related work.

Sr. No	Sensor Type	Materials	Fabrication Technology	References
1	Humidity	PEDOT:PSS, Methyl red and graphene oxide	Ink-jet printing and spin coating	[4]
2	Humidity	Chitosan-CeO_2_-CdO nanocomposite	Pressing	[18]
3	Humidity and temperature	Amino anthraquinone	Thermal deposition	[5]
4	Humidity and illumination	Copper phthalocyanine	Vacuum thermal evaporation	[6]
5	Temperature and humidity	Chitosan–CuO–Fe_3_O_4_ nanocomposite	Pressing	[7]
6	Temperature and infrared photodetector	Graphene	Microfabrication	[12]
7	Temperature sensors	Reduced graphene oxide	Screen printing and air spray coating	[13]
8	Temperature	Graphene–graphene oxide hybrid films	LBL method	[14]
9	Paper-based electrochemical sensors	CNTs	Laser cutting, drop-casting, and origami	[15]

## Data Availability

Data will be available upon request.
